# P02-020 - CAPS in Turkish children: treatment with ANTI IL1

**DOI:** 10.1186/1546-0096-11-S1-A127

**Published:** 2013-11-08

**Authors:** FK Kara Eroglu, N Besbas, F Ozaltin, Y Bilginer, A Duzova, S Ozen

**Affiliations:** 1 Pediatric Rheumatology Department, Hacettepe University Faculty of Medicine, Ankara, Turkey

## Introduction

Criyopyrin associated periodic syndrome (CAPS) has a heterogenous presentation and in a number patients mutations can not be found. Here we present our initial results with CAPS patients.

## Case report

### Results

All of our patients had symptoms within the first 3 months of life. All had fever, urticaria and persistant labaratory inflammation. All except one patient had failure to thrive. Except for the one patient with Muckle Wells syndrome all had neurological features ranging from headache to convulsions, hydrocephalus, cognitive dysfunction.Two patients, one without a mutation, had hearing impairment. Two patients have diarrhea during attacks. All were started on anti IL1 , one patient who did not respond to anakinra was started to canakinumab and on the fourth dose he developed MAS. After MAS was subsided canakinumab was re-started and he continues the drug without further problems. Presently three patients are on anakinra and three are on canakinumab, all with normal acute phases and improved quality of life. One patient has associated Duchenne muscular dystrophy.

## Discussion

### Conclusions

Anti IL1 treatment is efficacious in CAPS patients. Somatic mutations may enlighten the mutation negative patients. Until then classification criteria are needed to guide pediatricians in diagnosis and treatment.

## Disclosure of interest

None declared.
